# Decoding the Conformational Selective Mechanism of FGFR Isoforms: A Comparative Molecular Dynamics Simulation

**DOI:** 10.3390/molecules28062709

**Published:** 2023-03-17

**Authors:** Mingyang Zhang, Miersalijiang Yasen, Shaoyong Lu, De-Ning Ma, Zongtao Chai

**Affiliations:** 1 Medicinal Chemistry and Bioinformatics Center, Shanghai Jiao Tong University School of Medicine, Shanghai 200025, China; 2 Department of Orthopedic Surgery, Zhongshan Hospital (Xiamen), Fudan University, Xiamen 361015, China; 3 Institute of Energy Metabolism and Health, Shanghai Tenth People’s Hospital, Tongji University School of Medicine, Shanghai 200072, China; 4 Department of Colorectal Surgery, The Cancer Hospital of the University of Chinese Academy of Sciences (Zhejiang Cancer Hospital), Hangzhou 310022, China; madeningsdu@163.com; 5 Department of Liver Surgery and Transplantation, Key Laboratory of Carcinogenesis and Cancer Invasion of Ministry of Education, Liver Cancer Institute and Zhongshan Hospital, Fudan University, Shanghai 200032, China; 6 Department of Hepatic Surgery, Shanghai Geriatric Cancer, Shanghai 201104, China

**Keywords:** fibroblast growth factor receptor, P-loop, molecular dynamics simulations, Markov state models, network analysis

## Abstract

Fibroblast growth factor receptors (FGFRs) play critical roles in the regulation of cell growth, differentiation, and proliferation. Specifically, FGFR2 gene amplification has been implicated in gastric and breast cancer. Pan-FGFR inhibitors often cause large toxic side effects, and the highly conserved ATP-binding pocket in the FGFR1/2/3 isoforms poses an immense challenge in designing selective FGFR2 inhibitors. Recently, an indazole-based inhibitor has been discovered that can selectively target FGFR2. However, the detailed mechanism involved in selective inhibition remains to be clarified. To this end, we performed extensive molecular dynamics simulations of the apo and inhibitor-bound systems along with multiple analyses, including Markov state models, principal component analysis, a cross-correlation matrix, binding free energy calculation, and community network analysis. Our results indicated that inhibitor binding induced the phosphate-binding loop (P-loop) of FGFR2 to switch from the open to the closed conformation. This effect enhanced extensive hydrophobic FGFR2-inhibitor contacts, contributing to inhibitor selectivity. Moreover, the key conformational intermediate states, dynamics, and driving forces of this transformation were uncovered. Overall, these findings not only provided a structural basis for understanding the closed P-loop conformation for therapeutic potential but also shed light on the design of selective inhibitors for treating specific types of cancer.

## 1. Introduction

Fibroblast growth factors (FGFs), which signal through FGF receptors (FGFRs), are critical regulators controlling a wide range of basic physiologic processes in human development, such as cell survival, growth, proliferation, differentiation, and migration [1,2,3]. FGFRs are a superfamily of receptor tyrosine kinases (RTKs) that activate the downstream MAPK, PI3K/AKT, and STAT pathways, consisting of FGFR1, FGFR2, FGFR3, and FGFR4 [4,5]. They contain approximately 800 amino acids, including an extracellular growth factor binding domain, a single-pass transmembrane domain, and an intracellular tyrosine kinase domain.

Compelling evidence indicates that FGFR aberrations have been linked to a wide variety of human diseases. Approximately 7% of malignancies have an FGFR aberration [6]. For example, amplification of FGFR1 and FGFR2 has been found in 17% of squamous non–small cell lung cancer (sqNSCLC) [7] and in 5–10% of gastric cancer [8], respectively. FGFR3 gene fusions (mainly FGFR3-TACC3) have been observed in glioblastoma multiforme (GBM) and bladder urothelial tumors [9,10]. Thus, FGFRs, especially their kinase domains, are considered potential therapeutic targets for a variety of cancers [11].

In recent years, a growing body of small molecule inhibitors targeting FGFRs has been reported as potential disease treatments (Table 1) [12,13,14,15,16,17,18,19,20]. However, the secondary structures of FGFRs are similar, and the vast majority of inhibitors bind to the conserved ATP-binding sites, resulting in the poor selectivity of inhibitors towards a specific FGFR isoform. This causes adverse drug reactions such as hyperphosphatemia and tissue mineralization [21,22]. Hyperphosphatemia, one of the most frequently reported side effects of pan-FGFR inhibitors, is mediated by blockade of FGF23-FGFR1 signaling in the kidney, upregulating production of the active form of 1,25-dihydroxy vitamin D [23,24,25]. Therefore, how to develop subtype-selective FGFR inhibitors remains challenging and deserves exploration to overcome the adverse drug reactions of traditional pan-FGFR inhibitors [26,27].

**Figure 1:**
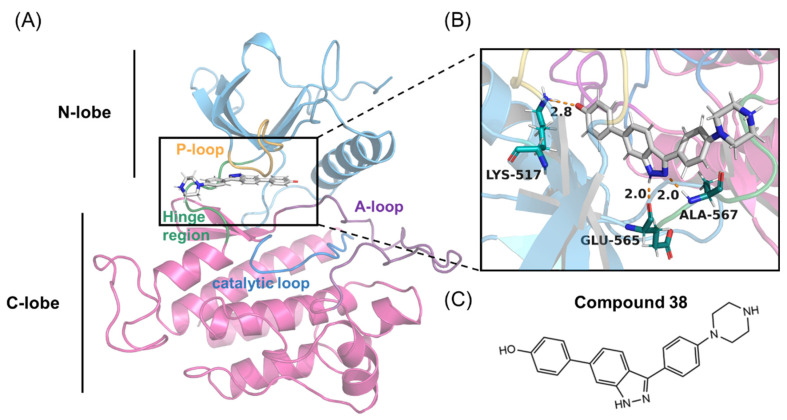
(**A**,**B**) Overall structure of the FGFR2 kinase domain in a complex with compound 38 (PDB code 7OZY). The N-lobe (blue) and C-lobe (pink), P-loop (orange), catalytic loop (marine), activation-loop (A-loop) (purple), hinge region (green), and compound 38 are highlighted. (**C**) Chemical structure of compound 38.

Recently, an indazole-based inhibitor (compound 38) has been discovered that exhibits markedly reduced inhibition towards FGFR2 compared to FGFR1 [28]. Compound 38 specifically inhibited FGFR2 with an IC_50_ of 29 nM, whereas it had an IC_50_ of 389 nM against FGFR1 and 758 nM against FGFR3, exhibiting a 13-fold selectivity preference for FGFR2 over FGFR1. Crystal structure analysis revealed that compound 38 has highly similar binding patterns with both FGFR1 and FGFR2 (Figure 1). However, the mechanism of this selective inhibition towards the FGFR2 isoform has not yet been elucidated.

Molecular dynamics (MD) simulations have become a robust tool for exploring the dynamic conformational changes at an atomic level and directly uncovering protein-ligand interaction mechanisms [29,30,31,32]. Here, extensive large-scale MD simulations combined with Markov state models (MSMs) were performed to investigate the selective inhibition mechanism of compound 38 towards FGFR1 and FGFR2. Our results indicated that compound 38 binding induced the phosphate-binding loop (P-loop) of FGFR2 rather than FGFR1 switching from the open to the closed conformations to form a hydrophobic channel. The key face-to-edge aromatic interaction between Phe492 and compound 38 stabilized the closed conformation of the P-loop in FGFR2. We further proposed a stepwise pathway for the P-loop transition, thus deepening our understanding of the P-loop folding mechanism. The present study provided the necessary structural insights into the mechanism of selective inhibition of FGFR isoforms and paved the way for the design of novel and potent isoform-specific inhibitors.

## 2. Results

### 2.1. Compound 38 Loading Enhanced Systems Stability

To elucidate the detailed selective inhibitory mechanism at the atomic level, we performed 1 μs × 5 length MD simulations of FGFR1 and FGFR2 in the apo and compound 38-bound states, leading to a cumulative simulation timescale of 20 μs. To evaluate the overall stability of simulation systems, root-mean-square-deviation (RMSD) analysis for all Cα atoms of FGFR was calculated (Supplementary Figure S1). For the equilibrated conformations, the RMSD values for the apo FGFR1, apo FGFR2, FGFR1-38, and FGFR2-38 systems were 3.71 ± 0.51 Å, 3.64 ± 0.43 Å, 3.02 ± 0.16 Å, and 2.78 ± 0.19 Å, respectively. It should be noted that the RMSD values for the apo systems were higher than those of inhibitor-bound systems, demonstrating the conformational flexibility of the apo systems. 

Next, the domain-specific influence of compound 38 binding was analyzed in terms of per-residue root mean square fluctuations (RMSF) (Supplementary Figure S2). The overall shape of the RMSF plots in different systems was generally similar. The flexible loops, such as the activation-loop (A-loop) and P-loop, have a higher RMSF. Typically, FGFR1-38 and FGFR2-38 systems displayed a lower RMSF, suggesting that the conformational dynamics were more stable in the presence of compound 38. Meanwhile, FGFR1-38 showed an overall higher RMSF compared to FGFR2-38, particularly in the A- and P-loops. A similar pattern was also found in the apo FGFR1 and apo FGFR2. Collectively, the highly flexible A-loop and P-loop may play a pivotal role in selective inhibition as transducers. In the following analysis, we mainly focused on these highly flexible regions.

### 2.2. Compound 38 Binding Enhanced Local Conformational Dynamics in FGFR2

**Figure 2:**
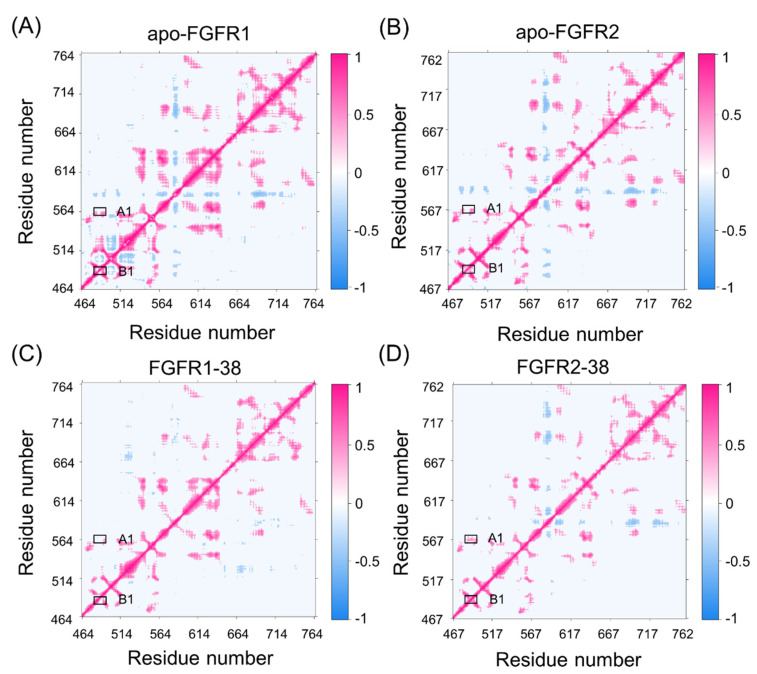
The dynamic cross-correlation matrices of apo-FGFR1 (**A**), apo-FGFR2 (**B**), FGFR1-38 (**C**), and FGFR2-38 (**D**). Color scales are shown at the right. The interactions whose absolute correlation coefficients are less than 0.3 are colored white for clarity.

To elucidate the different conformational dynamics of FGFR1 and FGFR2 triggered by compound 38, dynamic cross-correlation matrices (DCCMs) of Ca atoms were calculated and shown in Figure 2. The diagonal regions correspond to the movements relative to the residues themselves, while the other regions represent the movements between different residues. According to the color-coded modes, the pink indicates correlated motions between residues, while the blue indicates anti-correlated motions between residues. 

Overall, compound 38 binding dampened FGFR1 and FGFR2 correlations. In detail, A1 represented the correlated movement between the P-loop and the hinge region, and B1 represented the correlated movement within the P-loop. Both A1 and B1 represented the motions of the catalytic site. In the FGFR2-38 complex, there were enhancements of correlated movements between the P-loop and hinge region, whereas in the FGFR1-38 complex, these motions were negligible. Moreover, the P-loop showed an enhanced correlation in the FGFR2-38 complex compared with the FGFR1-38 complex. Differences in these correlations may be related to the distinct inhibitory activity of the inhibitor, because the P-loop is highly flexible and may play a vital role in binding affinity. This result suggested that the correlated movement of the P-loop may favor the binding of compound 38 to FGFR2. 

**Figure 3:**
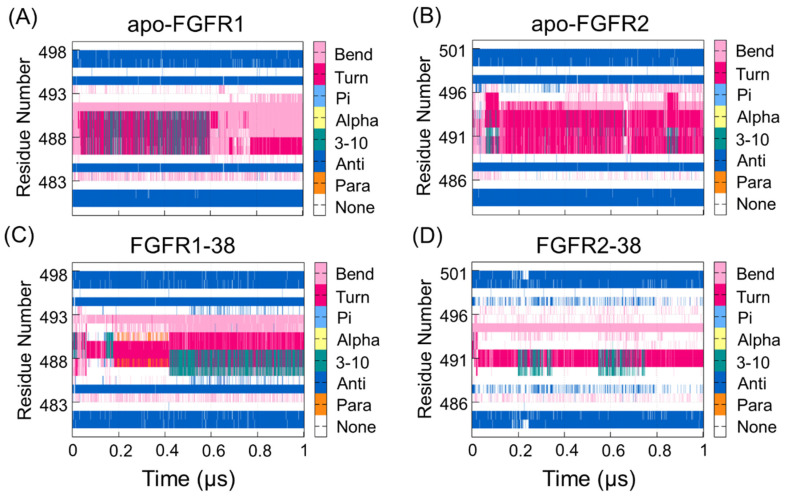
The DSSP analysis for residues during the simulation of the apo-FGFR1 (**A**), apo-FGFR2 (**B**), FGFR1-38 (**C**), and FGFR2-38 (**D**). The structure category corresponding to its colors is shown on the right. The figures were drawn by Gnuplot.

Given that the P-loop was highly flexible, there were enhanced correlations between the P-loop and hinge region. To further reveal the different real-time secondary structures of the P-loop in the FGFR1 and FGFR2 complexes, the defined secondary structure of proteins (DSSP) method was used to analyze the secondary structural elements of the P-loop (Leu484−Gln491 in FGFR1 and Leu487−Gln494 in FGFR2) and the surrounding β1- and β2-strands. As shown in Figure 3, the secondary structure of the P-loop became more disordered and unstable in response to ligand binding. Remarkably, the secondary structure of the P-loop in the FGFR2-38 complex showed a conformational transition from the β-turn and β-bend to the unstructured loops. The P-loop adopted the most disordered conformation in the FGFR2-38, which was in accordance with the DCCM observation (Figure 2D). The conformational changes indicated the enhanced conformational dynamics of the P-loop in response to ligand binding. This may play an important role in inhibitor binding selectivity and specificity, which has also been found in other similar kinases [33,34].

### 2.3. Compound 38 Binding Induced the Approaching Conformations of the N- and C-lobes in FGFR2

**Figure 4:**
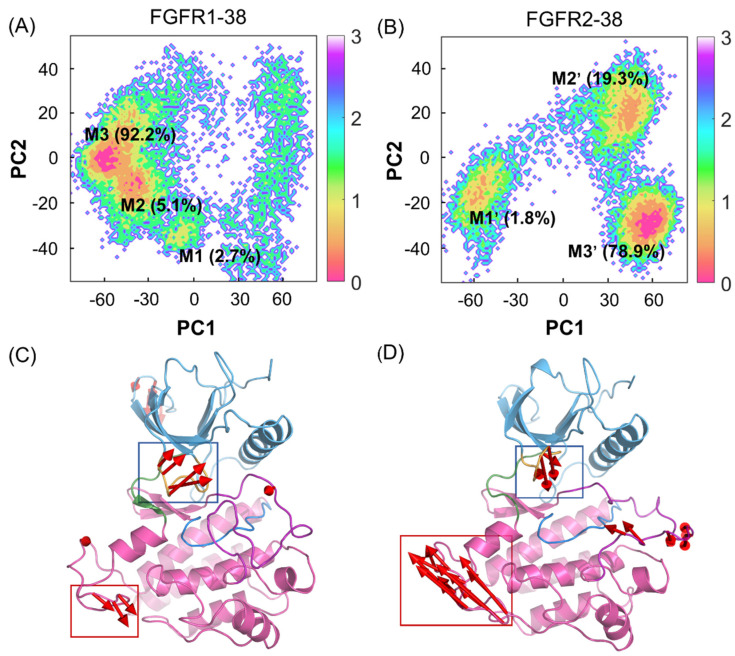
PCA of FGFR1-38 and FGFR2-38 systems. The free energy landscapes of the first and second principal components (PC1 and PC2) for the FGFR1-38 (**A**) and FGFR2-38 (**B**) systems. The unit of free-energy values is kcal/mol. The porcupine plots of the FGFR1-38 (**C**) and FGFR2-38 (**D**) systems depict the principal pattern of motion along PC1. The N-lobe (blue) and C-lobe (pink), P-loop (orange), catalytic loop (marine), A-loop (purple), and hinge region (green) are highlighted. The sizes and lengths of the red arrows are proportional to the amplitude of motions.

To unravel the dominant collective motions among different complexes, a principal component analysis (PCA) of the overall protein backbone was performed to characterize and compare the FGFR1-38 and FGFR2-38 systems. The two most dominant collective principal components (PC1 and PC2) were used to project the overall conformational landscape onto a two-dimensional (2D) diagram. As shown in Figure 4A,B, FGFR1 and FGFR2 had broadly distributed populations upon compound 38 binding. However, they adopted distinct dominant conformations. These predominant basins contained the majority of the simulation snapshots, mainly located at M3 (−59, 0) and M1′ (57, −28), respectively, which were well distinguished along PC1. 

To graphically visualize the dominant motions of different regions in FGFR, PC1 was projected onto the initial structure for each system (Figure 4C,D). In the FGFR1-38 and FGFR2-38 systems, the N- and C-lobes showed anticorrelated motions, but in opposite directions. Further insights into the domain dynamics along PC1 reflected the departing and approaching motions of the N- and C-lobes. These motions are characterized mainly by the relocation of the P-loop, which was considered a “breathing motion,” opening or closing the active site cleft [35]. The hinge region acted as a bridge between the two lobes. Remarkably, in the presence of compound 38, FGFR2 exhibited the approaching conformations, while FGFR1 exhibited the distinct departing conformations. This suggested that the overall approaching conformation contributed to ligand binding affinity. Thus, compound 38 can induce the conformational ensemble of FGFR2 into a different state from FGFR1, which implies a conformational selective mechanism. Meanwhile, PC1 can be considered a significant parameter to measure the ability of compound 38 to bind to FGFR.

### 2.4. Compound 38 Binding Induced a Conformational Transition from the Open to the Closed Conformation of the P-loop in FGFR2 

Since PCA only revealed the dynamic and structural coupling between the N- and C-lobes in the FGFR2 to quantify the crucial role of the P-loop in the binding process of compound 38, two collective variables were further chosen to delineate and analyze the free energy landscapes. One proper collective variable is the RMSD of the P-loop, referring to the starting structure exhibiting the structural alterations towards the closed state. The other is the distance between the P-loop and compound 38, which characterizes the displacement of the P-loop shifting between the open and closed conformations.

**Figure 5:**
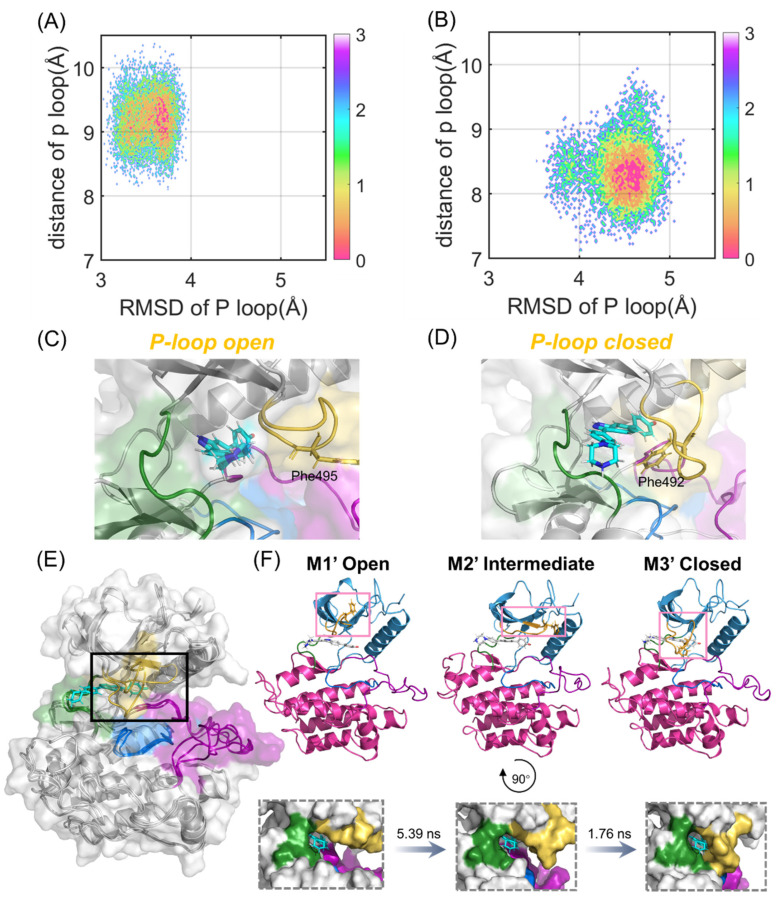
P-loop conformational analysis. Free-energy landscapes of FGFR1-38 (**A**) and FGFR2-38 (**B**) systems based on the RMSD and distance of the P-loop. The most representative structure in the ligand-binding domain of FGFR1-38 (**C**) and FGFR2-38 (**D**). The P-loop (orange), catalytic loop (marine), A-loop (purple), and hinge region (green) are highlighted. Phe495 in FGFR1 and Phe492 in FGFR2 are shown as sticks. (**E**) Structural overlapping of the three most represented conformations (M1′-M3′) based on the PCA results in FGFR2-38. (**F**) The kinetic transition pathway of FGFR2 from the P-loop open state (M1′) to the P-loop closed state (M3′) through the intermediate state (M2′) is shown as a cartoon and on the surface, respectively. The transition timescales for the states are provided above the arrows.

According to Figure 5A,B, the energy basins of the FGFR1-38 and FGFR2-38 systems are distributed at (3.70 Å, 9.23 Å) and (4.56 Å, 8.22 Å), respectively. The FGFR2-38 system exhibited a higher RMSD value. Hence, the P-loop conformational landscapes were relatively more widely distributed over the free energy landscapes, which were consistent with the DSSP analysis. Notably, in the representative structure, the P-loop of FGFR1 was relatively loose and in an open conformation (Figure 5C), while the P-loop of FGFR2 was closer to compound 38 and in a closed conformation (Figure 5D). In the light of Figure 5D, the P-loop extended inward, and the aromatic residue Phe492 on the tip of the P-loop flipped to the ATP-binding site, forming aromatic π-π stacking interactions with the inhibitor. This led us to infer that the inward movement of the P-loop in the FGFR2-38 complex promoted the binding of the inhibitor to the protein. Based on previous studies, despite being rare, similar P-loop closed conformations have been found in some kinase-inhibitor complexes to accommodate the substrate [36,37,38]. Statistical analyses of the crystal structure of kinases indicated that inhibitors able to induce the P-loop closed conformation tend to be more selective and highly potent [33]. Subsequently, Collie and coworkers reported a virtual screen for c-MET kinase and discovered a series of inhibitors that bind to the P-loop closed conformation of the protein [39].

To further explore the key conformational intermediate states of P-loop folding in the FGFR2-38 complex, we used PyEMMA to construct Markov state models based on PCA data. They were proven to be Markovian through the implied timescale test and the Chapman−Kolmogorov test (Figure S3) [40]. Conformational ensembles were clustered into three MSM metastable states, and the most representative conformations from the metastable states were extracted to examine their structural properties. The mean first passage times (MFPTs) of MSMs were calculated to probe the kinetics along the P-loop folding transition. 

As shown in Figure 5E, the P-loop conformation changed largely during the simulations in the FGFR2. From M1′ to M3′, the P-loop transformed from the open conformation to the closed conformation in response to compound 38 binding (Figure 5F), which was in agreement with our conformational landscape analysis. The MFPTs analysis indicated that FGFR2 transformed along the path M1′-M2′-M3′. The P-loop of the first state M1′, especially Phe492, was in an extended state. In contrast, the P-loop of M2′ moved inward, and the angle of benzene in Phe492 was twisted to a certain extent, implying that M2′ was in a key intermediate metastable state. In the M3′ state, the P-loop was completely folded, and the sequential conformational transition from the open to the closed conformations was achieved. The transition time from M1′ to M2′ was significantly longer than that from M2′ to M3′, indicating that the rate-determining step of the P-loop transition was the extension of P-loop from the initial to intermediate states. Collectively, these results indicated that the association of FGFR2 with compound 38 led to the prominently approaching characteristics of the N- and C-lobes. Moreover, compound 38 triggered the inward relocation of the P-loop, ultimately resulting in increased binding affinity and inhibitory activity. 

### 2.5. The Hydrophobic Channel by the Closed P-loop Was a Major Mechanism for Selective Inhibition 

**Figure 6:**
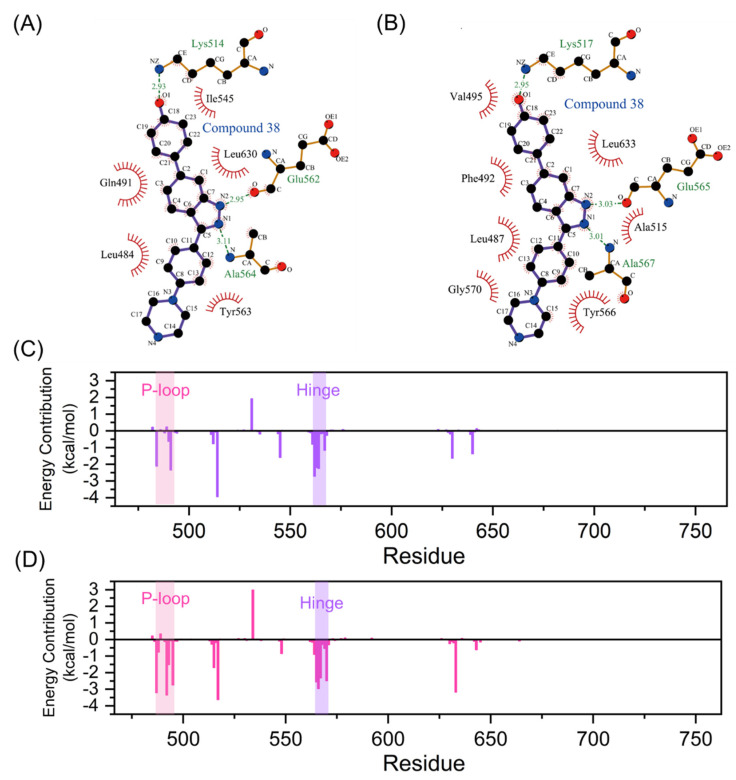
Intermolecular forces between FGFR and compound 38. Hydrogen bonds and hydrophobic interactions of FGFR1-38 (**A**) and FGFR2-38 (**B**) complexes are visualized by LigPlot+. Hydrogen bonds and hydrophobic interactions are shown as green dashed lines and red arcs, respectively. Residue contributors to the binding free energy of compound 38 to FGFR1 (**C**) and FGFR2 (**D**) calculated by the MM-GBSA method. The P-loop and hinge region are marked with pink and purple backgrounds, respectively.

The intermolecular interactions, such as hydrogen bonds, ionic interactions, and hydrophobic interactions, serve important roles in the protein-ligand interactions [41]. To uncover contributions of the folded P-loop to the selectivity, the intermolecular interfaces in the most representative structures of FGFR1-38 and FGFR2-38 were analyzed and depicted in Figure 6A,B with the help of Ligplot+ [42]. A comparative analysis of FGFR1-38 and FGFR2-38 complexes suggested that the tight binding of the inhibitor was due to the key complementary H-bond interactions and hydrophobic interactions. As a binding scaffold, the indazole moiety formed two hydrogen bonds with the hinge backbone (Glu565 and Ala567), and the phenol oxygen atom formed a hydrogen bond with Lys517. A summary of hydrogen bond occupancy was shown in Tables S1 and S2.

Hydrophobic interactions differed significantly between the FGFR1-38 and FGFR2-38 systems (Figure 6A,B). Owing to the folded P-loop conformation (Figure 5D), the hydrophobic residues in the P-loop, such as Leu487 and Phe492, approached Tyr566 in the hinge region and Leu633 in the A-loop to create a hydrophobic tunnel around the inhibitor as well as enhance communications between the N- and C-lobes in the FGFR2-38 system. Owing to the open P-loop conformation (Figure 5C), the Phe492 was protruded into the solvent and had no interactions with the inhibitor and other hydrophobic residues in the FGFR1-38 system, which was markedly different from the closed P-loop state in the FGFR2-38 system.

Based on the above analysis, we hypothesized that hydrophobic interactions may be the key factor for the selective stabilization of the FGFR2-38 binding pattern. To test this hypothesis, the total binding free energy of compound 38 towards FGFR1 and FGFR2 was estimated using the MM-GBSA method and decomposed into residues to obtain the detailed contributions of separate residues [43]. The total energetic component contributions were summarized in Table 2. The binding free energies for FGFR1-38 and FGFR2-38 were −30.86 ± 3.73 kcal/mol and −36.64 ± 4.39 kcal/mol, respectively. Obviously, the van der Waals interactions played a dominant role in the selective binding of the inhibitor to FGFR2 (−18.60 kcal/mol for FGFR1 vs. −46.93 kcal/mol for FGFR2). Therefore, the specific binding of compound 38 to FGFR2 was primarily controlled by van der Waals interactions rather than electrostatic interactions.

Here, the energy contribution of residues is plotted in Figure 6C,D. Residues that contributed more than the 1 kcal/mol threshold were considered significant for binding and shown in Table S3. Among them, the interfacial residues involved in hydrogen bonds and hydrophobic interactions (Figure 6A,B) substantially contributed to the binding free energy. These residues were mainly distributed in the P-loop, the hinge region, and the catalytic loop, demonstrating that these highly flexible loops played a significant role in inhibitor binding. Of note, Phe492 from P-loop had clearly more contribution in the FGFR2-38 complex compared to the FGFR1-38 complex (−3.35 vs. 0.23 kcal/mol), consistent with our analysis that the binding of compound 38 induced FGFR2 to form hydrophobic channels. Furthermore, Leu487, Gly493, and Val495 from the P-loop and Tyr566 and Gly570 from the hinge region also showed more contribution (Table S3) in the FGFR2-38 complex because of the approaching shift of the N- and C-lobes in the FGFR2. Thus, the MM/GBSA results further confirmed our analysis of extensive hydrophobic contacts. Hydrophobic residues primarily contributed to the selective binding of compound 38 to FGFR2. The presence of the conserved residue Phe492 seemed to be critical for the stabilization of the P-loop folded structure via π-π stacking and hydrophobic interactions with the ligand [33]. Compound 38 could induce FGFR2 to adopt a P-loop folded conformation, which was more stable due to the key hydrophobic interactions of Phe492. In conclusion, energy decomposition analysis showed important interaction residues and provided guidelines for further inhibitor optimization.

Collectively, the P-loop conformation can be used as an efficient regulatory mechanism in RTK regulation. Kinase inhibitor compound 38 can specifically perturb the dynamic conformational ensemble of FGFR2 and stabilize the closed P-loop conformation. In this unusual closed P-loop conformation, the binding site adopts a tunnel-like shape that provides a significant hydrophobic enclosure to the inhibitor and shields it from the solvent (Figure 5D) [37].

### 2.6. The Interaction Network Reveals the Driving Force of P-loop Folding 

**Figure 7:**
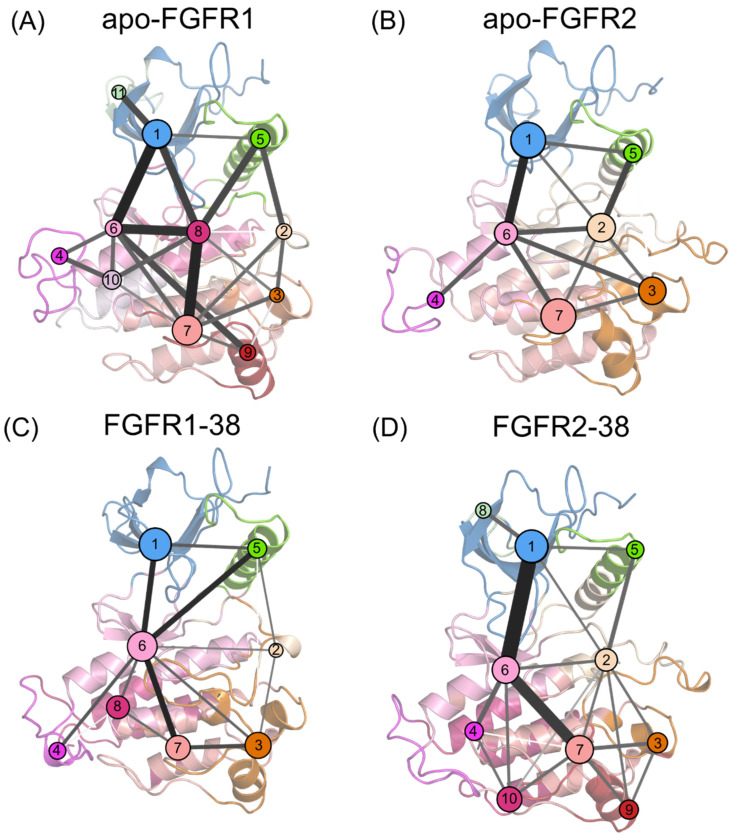
Community networks superimposed on the 2D structure of the apo-FGFR1 (**A**), apo-FGFR2 (**B**), FGFR1-38 (**C**), and FGFR2-38 (**D**) systems. Each circle represents an individual community. The size of the circle is proportional to the number of residues in each community, while the widths and colors of the lines connecting communities represent the corresponding intercommunity connections.

The P-loop adopts a closed conformation that forms extensive contacts with the inhibitor and contributes to the selectivity of the FGFR2-38 complex. However, what drives the closed P-loop conformation remains unclear. The P-loop residues in FGFR2 are the same as those in FGFR1, which rules out the influence of endogenous P-loop factors. Therefore, it was suggested that the folding of the P-loop occurred under specific signal propagation conditions. To investigate the propagation pathways of signals within FGFR2 during the P-loop transition, community network analysis (CNA) was conducted based on the Girvan−Newman algorithm [44]. The variational coupling between the communities was quantitatively estimated in all simulated trajectories. For at least 75% of the time, residues within a 4.5-distance cutoff were grouped into the same communities, which were treated as synergistic functional units in the global protein structure. The visualized community composition and connections are shown in Figure 7.

From a global view, the topological features and the intercommunity communications of the four systems were similar. In the apo systems, there were 11 communities and 22 pathways in FGFR1, as well as 7 communities and 12 pathways in FGFR2. In the ligand-bound systems, there were 8 communities and 14 pathways in the FGFR1-38 complex, as well as 10 communities and 19 pathways in the FGFR2-38 complex. In FGFR1, upon compound 38 binding, community 7 was incorporated into community 6, community 9 was incorporated into community 3, and community 11 was incorporated into community 1. Compound 38 strengthens the community association within the N- and C-lobes and may thereby capture the ATP-binding pocket in an open state. In contrast, in the FGFR2-38 complex, communities 8, 9, and 10 were independent from communities 1, 3, and 7, respectively, implying that their signal linkages to surrounding residues were diminished. Overall, the FGFR2-38 complex showed more fragmented communities and an elevated complexity of community connectivity, suggesting that inhibitor binding reshaped the community topology, which may promote inhibitor binding and selectivity. 

In all complexes, community 1 was composed of most of the N-lobe, including the P-loop and the β1-5 sheets. Community 6 was situated at the interface between the N- and C-lobes, encompassing the hinge region, β6 and β7 sheets, and αD helix. Community 7 was composed of the αF and αH helices. Communities 1 and 7 are strongly connected to community 6, indicating extensive interactions between the N- and C-lobes. Particularly, these connections were markedly strengthened in the FGFR2-38 complex (Figure 7D), as represented by thicker intercommunity sticks, indicating enhanced signal flow between them. Hence, the P-loop allosteric signals were propagated via the αF and αH helices, the β6 and β7 sheets, and the hinge region to the P-loop. Among them, Community 6 served as a hub for signal transmission and participated in an essential part of the ATP binding pocket. It received regulatory signals from community 7 and further allosterically promoted them towards the distal P-loop (community 1). 

In summary, we proposed that compound 38 binding affected the ATP binding pocket through an enhanced information flow from the C- to N-lobes, thus promoting the transition of the P-loop from the open to the closed conformation and its crosstalk with the hinge region.

## 3. Discussion

FGFRs are crucial for the survival, growth, and maturation of tumor cells [1]. Due to the high structural similarity of the kinase domains in FGFRs, previous work mainly focused on the pan-FGFR inhibitors, which target multiple isoforms. However, targeting multiple FGFR isoforms may increase toxic side effects and limit the efficacy required for specific FGFR inhibition. Therefore, the study of isoform-selective inhibitors and their inhibitory mechanisms is an emerging research field that is germane to the design of novel and potent isoform-selective inhibitors. 

Here, we revealed the isoform-selective inhibitory mechanism by which compound 38 induced the P-loop closed conformation in FGFR2 through multiple, large-scale MD simulations. Overall, FGFR1-38 and FGFR2-38 complexes exhibited fewer fluctuations compared with the apo proteins, suggesting more stable conformational dynamics. Locally, upon compound 38 binding, the movement and the secondary structure of the P-loop became more intense and disorganized, as revealed by the DCCM and DSSP analyses. PCA analysis unveiled the large-scale departing and approaching motions between the N- and C-lobes in the FGFR1-38 and FGFR2-38 complexes, resulting in the open and closed conformations of the catalytic cleft, respectively. Next, comparison of the conformational free energy landscape of the P-loop between FGFR1-38 and FGFR2-38 systems revealed that the closed conformation of the P-loop contributed to the selective binding of compound 38 to FGFR2. Furthermore, the PCA free-energy landscape was identified and confirmed by MSM, and the representative conformations of the intermediates were extracted. The stepwise transition pathway for the P-loop from the open to closed conformations was elucidated, deepening our understanding of the P-loop folding mechanism. This structural transition could shift the position of Phe492, thereby facilitating stabilization through hydrophobic interactions with the inhibitor. Moreover, MM/GBSA analysis revealed that the binding free energy of the FGFR2-38 complex was lower than that of the FGFR1-38 complex, mainly due to the increased favorable contribution from the van der Waals interactions. More specifically, we identified that the hydrophobic residues played significant roles in the inhibitor binding using the decomposition of free energy and intermolecular interface analysis, confirming our hydrophobic channel hypothesis. Finally, the visualized community network clearly delineated the pathway and intensity of the regulatory crosstalk in each system, revealing that the long-distance signal flow from the C- to N-lobes in the FGFR2-38 complex led to the P-loop folding via a communication hub in the hinge region.

Many kinases can adopt a closed P-loop conformation [37,39]. Drugs designed for different kinase conformations, such as DFG-out [45,46] and αC-helix-out [47,48], have significant therapeutic value. From the perspective of molecular design, the present study provides guidance for optimizing FGFR subtype-selective inhibitors. First, the apparent preference of the closed P-loop conformation can be exploited in the development of selective inhibitors for FGFR2 and other kinase targets amenable to this binding mode. Second, intermolecular interactions such as hydrogen bonds and hydrophobic interactions are critical for stabilizing protein-ligand complexes. Well-designed inhibitors are supposed to form extensive hydrophobic interactions with the protein binding site, thereby increasing the stability of the complex. Third, binding free energy analysis can be used to selectively replace residues hindering interactions and strengthen those facilitating interactions. Above all, the in-depth mechanisms underlying the conformational selectivity of inhibitors to FGFR isoforms may provide important information for designing novel isoform-selective inhibitors as well as contribute to the solution of adverse drug reactions associated with pan-FGFR inhibitors.

Another strategy for the design of selective FGFR inhibitors is to apply allosteric effects. Allostery refers to the effect of allosteric modulators by interacting with sites that are spatially and topographically distinct from the functional site. The residue diversity of allosteric sites in a FGFR subfamily allows for the development of allosteric modulators with higher selectivity and better physio-chemical properties, which offers a new paradigm for subtype-selective drug design [49].

## 4. Materials and Methods

### 4.1. Preparation of Stimulation Systems

The crystallographic structures of FGFR1 in a complex with compound 38 (henceforth FGFR1-38) (PDB ID: 7OZB) and FGFR2 in a complex with compound 38 (henceforth FGFR2-38) (PDB ID: 7OZY) were downloaded from the RCSB Protein Data Bank (PDB). By deleting compound 38, we also constructed the apo FGFR1 and apo FGFR2 systems for the reference. Before simulations, UCSF Chimera and Pymol were utilized to remodel the missing non-terminal residues of the protein to ensure integrity and remove nonprotein co-crystallized molecules (sulfate ion and ethanediol) from the crystal structures of the protein. The C488A and C584S mutations of the FGFR1-38 complex (PDB ID: 7OZB) were changed back to the wild-type residues using UCSF Chimera.

### 4.2. Molecular Dynamics Simulations

To elucidate the protein dynamics in a real environment, MD simulations were performed on the apo FGFR1, apo FGFR2, and FGFR1-38 and FGFR2-38 systems using the AMBER 18 program. The force field parameters for the protein and compound 38 were obtained using the ff14SB force field and the antechamber package [50,51]. The LEaP module was used to solvate each system in a TIP3P explicit water cube of 10 Å length, followed by addition of counter-ions to maintain the neutralization. To build a physiological environment, 0.154 mol/L NaCl was randomly added to the system. The GPU-accelerated version of PMEMD was used for all MD simulations [52]. In the process, the steepest descent and conjugate gradient energy minimization methods were used to eliminate spatial conflicts. All four solvated systems were minimized by a total of 150,000 steps in two rounds of energy minimization, first allowing nonprotein molecules to move (50,000 steps) and then allowing all atoms to move without any restriction (100,000 steps). After minimization, all systems were then gradually heated from 0 to 300 K within 300 ps using the Langevin dynamics algorithm in a canonical ensemble (NVT) and equilibrated for 700 ps. Finally, five replicas of 1 μs MD simulation in an isothermal isobaric ensemble (NPT) at 1 atmospheric pressure (atm) and 300 K were carried out, whose integral step was set to 2 fs. The Particle Mesh Ewald (PME) method and SHAKE algorithm were conducted to calculate the long-range electrostatic interactions with a 10 Å cutoff and constrain all the covalent bonds involving hydrogen atoms, respectively [53,54]. PyMol version 1.3 was used for visualization and rendering.

### 4.3. Dynamic Cross-Correlation Matrix (DCCM) Analysis

The dynamic cross-correlation map (DCCM) analysis, which was performed using the CPPTRAJ module of AMBER18 on all systems [55,56], represented the correlation between different residues. The element cross-correlation coefficients $C_{ij}$ of normalized cross-correlation matrix C were calculated using the following equation:

$$C_{ij}=\frac{\langle \Delta r_{i}\cdot \Delta r_{j}\rangle }{{\left(\langle \Delta r_{i}^{2}\rangle \langle \Delta r_{j}^{2}\rangle \right)}^{1/2}}$$

In this formula, $\Delta r_{i}$ and $\Delta r_{j}$ indicate the displacement vectors of the *i*th and *j*th Cα atomic coordinates, respectively.

### 4.4. Principal Component Analysis and Free Energy Landscapes

To clearly describe the dominant protein dynamics and explain the phenomena of interest, PCA has been widely applied to simulation systems. As a systematic statistical method, PCA reduced the original large number of dependent variables (atomic coordinates) to smaller and independent sets [57,58]. By solving the eigenvalues and eigenvectors of the covariance matrix, the motions needed to be observed were transformed into independent principal components (PCs), the linear combinations of interrelated variables, by the following equation:

$$PC_{i}=\alpha_{i1}x_{1}+\alpha_{i2}x_{2}+\ldots +\alpha_{im}x_{m}=\overset{m}{\underset{j=1}{\sum }}\alpha_{ij}x_{j}$$

Among these vectors, the first two principal components (PC1 and PC2) often accounted for enough of the variance to provide the dominant motions during the MD simulations [59]. During PC analysis, for a better comparison with different simulations, we performed RMS-fits to the same initial structure to exclude translational and rotational motions of proteins. 

The free energy landscape (FEL) is an effective tool to explore the conformational ensembles of proteins [29,57], which is constructed using the following equation:

$$G_{i}=-k_{B}T\ln\left(\frac{N_{i}}{N_{m}}\right)$$

where $k_{B}$ is the Boltzmann constant and *T* represents the temperature of the simulation system. $N_{i}$ and $N_{m}$ are the populations of the *i*th and most populated bins, respectively. For this work, we chose the first two components (PC1 and PC2), RMSD values, and distances as the reaction coordinates to build the FELs. Energy levels were represented through color-coded modes. 

### 4.5. Binding Free Energy

The MM/GBSA (Molecular Mechanics/Generalized Born Surface Area) method packaged in AMBER 18 was used to quantify the total binding free energy and per-residue decomposition of equilibrated MD simulation trajectories [43,60]. In this method, the binding free energies, which were made up of binding free energy in vacuum ($\Delta G_{bind,vacuum}$) and solvation free energy ($\Delta G_{solv}$), were estimated from the following sum:

$$\begin{array}{cc} \Delta G_{bind,solve} & =\Delta G_{bind,vacuum}+\Delta G_{solv}\\ & =\Delta E_{MM}-T\Delta S+\Delta G_{solv}\\ & =\Delta E_{internal}+\Delta E_{elec}+\Delta E_{vdW}+\Delta G_{pol}+\Delta G_{np}-T\Delta S \end{array}$$

where the first three terms are standard molecular mechanics energy terms ($\Delta E_{MM}$) from internal (bond, angle, and dihedral), electrostatic, and van der Waals interactions. $G_{pot}$ and $G_{np}$ are the polar and non-polar contributions to the solvation-free energies ($\Delta G_{solv}$). In addition, due to the inaccuracy of conformational entropy estimated by normal mode analysis and the structural similarity between FGFR1-38 and FGFR2-38 systems, the entropy term (TΔS) was ignored in our calculation [61]. 

### 4.6. Markov State Model Construction and Validation

Markov state models (MSMs) are a powerful mathematical framework to examine the key conformational states during MD simulation [29,62]. Through the Python library PyEMMA, PCA data of the overall protein backbone in FGFR1-38 and FGFR2-38 systems was taken as input to build Markov state models and verify the analysis [40,63]. First, we used the implied timescale (ITS) test to validate the Markovianity of models:

$$t_{i}=\frac{-\tau }{\ln\left|\lambda_{i}\left(\tau \right)\right|}$$

with $\lambda_{i}$ being the eigenvalues of the Markov transition matrix and *τ* being the lag time. The K-means clustering algorithm was used to decompose the free-energy landscape into microstates. Next, MSMs for FGFR1-38 and FGFR2-38 systems were constructed. We scanned a series of K numbers, maximum iteration numbers, and lag times in order to select the “best” MSMs (i.e., the implied time scales are lag-time independent and the longest among the MSMs). The Markovianity was examined through the ITS test yielded from the corresponding MSM. After careful selection, we chose a lag time of 3 ns and the maximum iteration number of 100 with 100 centers. Then, the Perron Cluster Analysis (PCCA++) algorithm was applied to coarse-grain the obtained microstates into metastates, whose Markovianity was further confirmed by the Chapman–Kolmogorov test:

$$T\left(n\tau \right)=T{\left(\tau \right)}^{n}$$

where T(nτ) is the transition probability matrix at time *nτ* and τ is the lag time. Finally, the MDTraj package was used to select the representative conformation of each metastate according to the similarity score S_ij_ [64]. The mean first passage times (MFPTs) between two states i and j (MFPTi→j) were calculated based on the constructed transition probability matrix.

### 4.7. Community Network Analysis

With the help of the NetworkView plugin in VMD, we calculated the correlation coefficient matrix $C_{ij}$ to analyze intercommunity interactions and the community network [65]. The C*α* atoms of the FGFR were considered to be a set of nodes representing their corresponding residues [66,67]. Edges were defined between nodes within a 4.5 Å distance cutoff for at least 75% of the simulation time and calculated by

$$d_{i,j}=-\log\left(\left|C_{i,j}\right|\right)$$

where *i* and *j* represent the two nodes. Embedding the Girvan−Newman divisive algorithm, we applied the gncommunities program to generate the substructure of communities [68]. We gauged the intercommunity connectivity by the betweenness value. Furthermore, communities with fewer than three residues were dropped. 

## Figures and Tables

**Table 1:** Representatives of pan-FGFR inhibitors that target FGFRs.

Inhibitor	Targets	Disease Type	Reference
AZD4547	FGFR1–3	ER+ breast cancer	[12]
Derazantinib (ARQ-087)	FGFR1–4	Advanced intrahepatic cholangiocarcinoma with FGFR2 gene aberrations	[13]
Erdafitinib (JNJ42756493)	FGFR1–4	Locally advanced or metastatic bladder cancer, etc.	[14]
Futibatinib (TAS-120)	FGFR1–4	Metastatic breast cancer with FGFR2 amplification	[15]
Infigratinib (BGJ398)	FGFR1–3	Advanced or metastatic cholangiocarcinoma	[16]
LY2874455	FGFR1–4	Advanced cancer	[17]
Pemigatinib (INCB054828)	FGFR1–3	Unresectable or metastatic cholangiocarcinoma	[18]
Rogaratinib (BAY1163877)	FGFR1–3	Squamous non-small cell lung cancer	[19]
Zoligratinib (Debio-1347)	FGFR1–3	Advanced solid tumors	[20]

**Table 2:** Binding free energies (kcal/mol) of FGFR1-38 and FGFR2-38 systems computed by the MM-GBSA method.

Complex	*ΔE_ele_*	*ΔE_vdw_*	*ΔG_pol_*	*ΔG_np_*	*ΔG_binding_*
FGFR1-38	−47.72 ± 3.00	−18.60 ± 7.85	41.28 ± 6.18	−5.82 ± 0.25	−30.86 ± 3.73
FGFR2-38	−40.92 ± 3.09	−46.93 ± 9.89	56.48 ± 8.03	−5.27 ± 0.29	−36.64 ± 4.39

*ΔE_ele_*: electrostatic energy; *ΔE_vdw_*: Van der Waals energy; *ΔG_pol_*: polar contribution to solvation; *ΔG_np_*: non-polar contribution to solvation; *ΔG_binding_*: total binding free energy.

## Data Availability

Not applicable.

## Supplementary Materials

The following supporting information can be downloaded at: https://www.mdpi.com/article/10.3390/molecules28062709/s1, Figure S1: Root-mean-square deviations (RMSD) of all systems during simulation, Figure S2: Root-mean-square fluctuations (RMSF) of all systems during simulation, Figure S3: Implied timescale test of all constructed MSMs and Chapman-Kolmogorov test of metastable states, Table S1: Percentage of hydrogen bonding interactions in FGFR1-38 complex, Table S2: Percentage of hydrogen bonding interactions in FGFR2-38 complex, Table S3: Residues contributed more than 1 kcal/mol in each system.
