# Supplementary material for: Decoding the Conformational Selective Mechanism of FGFR Isoforms: A Comparative Molecular Dynamics Simulation
Source: Molecules. 2023 Mar 17;28(6):2709. doi: 10.3390/molecules28062709 (PMC10052029; doi:10.3390/molecules28062709)
Supplement: Supplementary file 1 [file molecules-28-02709-s001.zip › molecules-2262945-supplementary.pdf]

Supplementary information

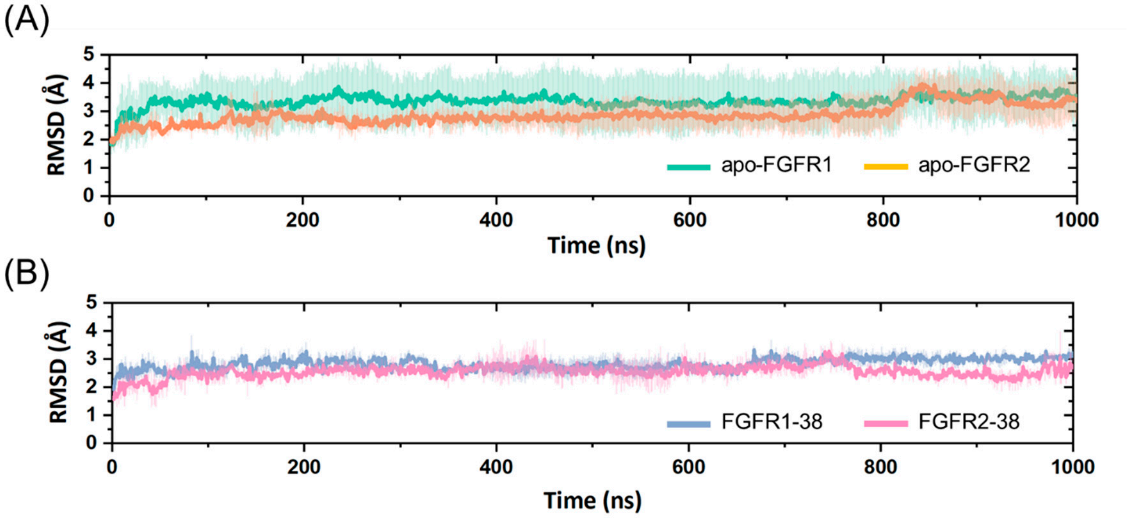

Figure S1: Root-mean-square deviation (RMSD) analysis for 1µs based on the Cα atoms of FGFR1 and FGFR2 with respect to the initial structure. The SEM error bars show RMSD for 5 replicas related to the average value.

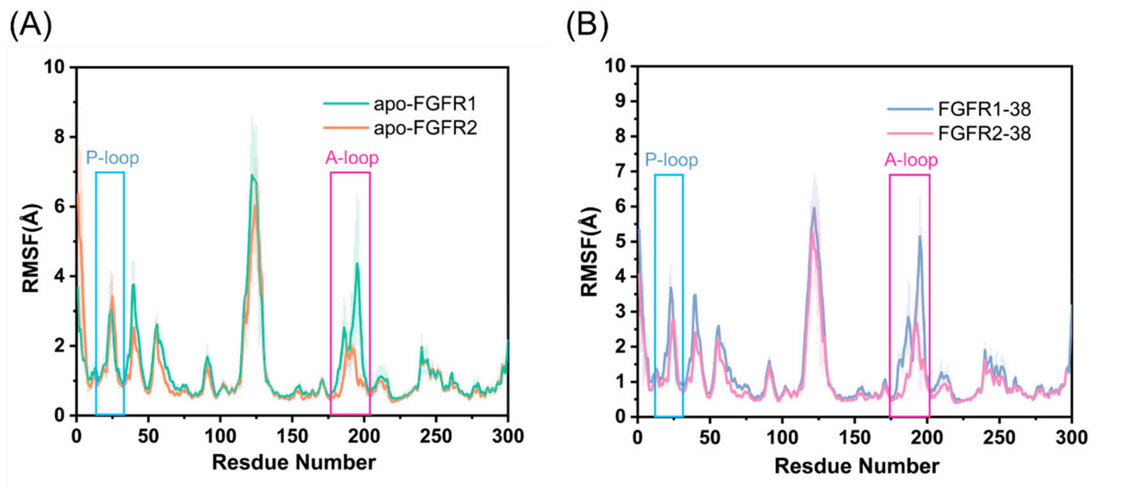

Figure S2: Root-mean-square fluctuations (RMSF) analysis for 1µs based on the Cα atoms of FGFR1 and FGFR2 with respect to the initial structure. The SEM error bars show RMSF for 5 replicas related to the average value.

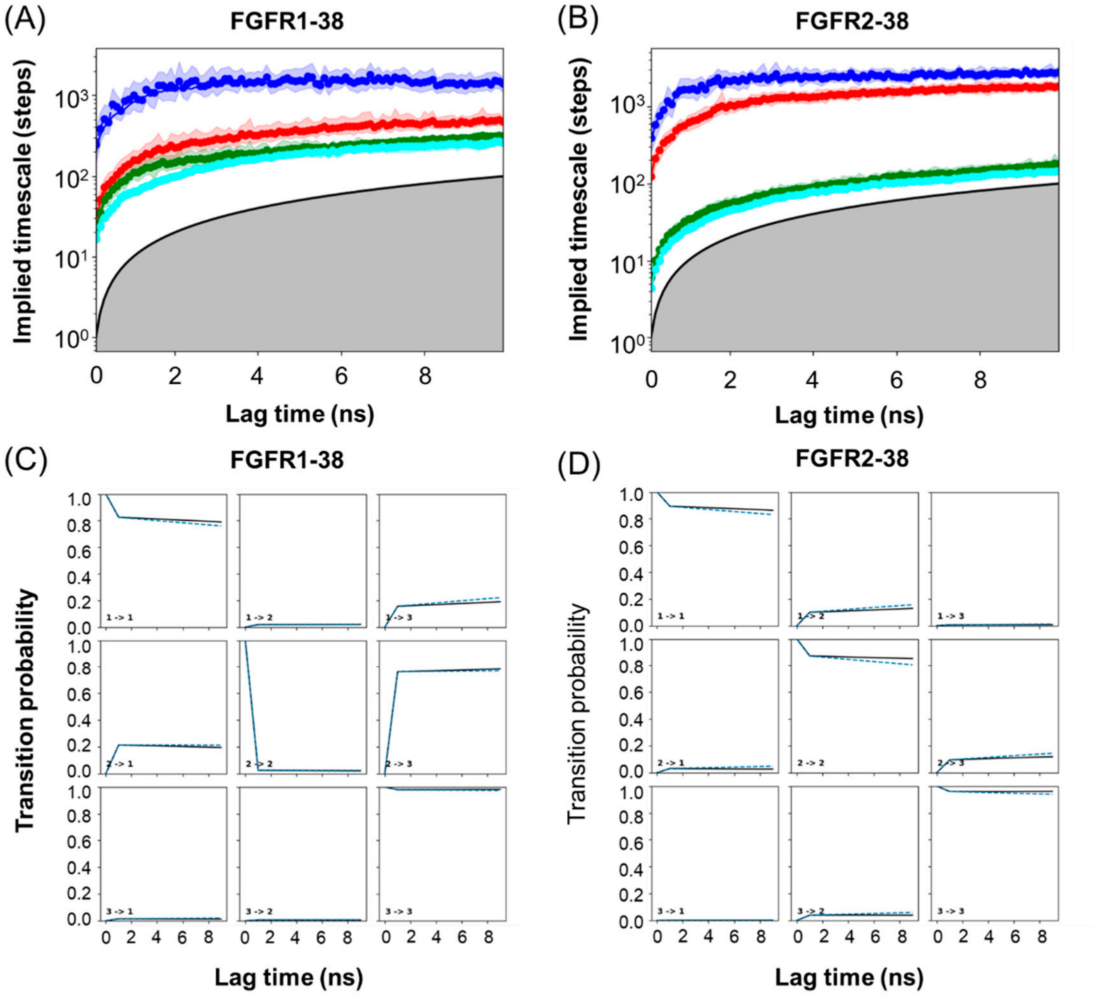

Figure S3: Implied timescale test for MSMs in FGFR1-38 (A) and FGFR2-38 (B) at different lag times. Blue, red, green, and cyan lines show the timescale τ1, τ2, τ3 and τ4. Black line represents x=y in logarithmic coordinates, which separates the area where the dynamics of the processes is resolvable (white) from the non-resolvable area (grey). Chapman-Kolmogorov test of metastable states for FGFR1-38 (C) and FGFR2-38 (D) systems. The black solid estimate lines are the transition probability predicted by MSMs, while the blue dotted predict lines are practical transition probability observed in trajectories.

Table S1: Key hydrogen bonding interactions in FGFR1-38 complex

Acceptor	Donor	Frac
Glu562	38	80.97%
38	Lys514	51.78%
38	Ala564	19.06%

Table S2: Key hydrogen bonding interactions in FGFR2-38 complex

Acceptor	Donor	Frac
Glu565	38	69.87%
38	Lys517	56.67%
38	Ala567	40.03%

Table S3: The significant residues contributed more than 1 kcal/mol in compound 38 binding to FGFR1 and FGFR2 complexes, respectively.

Residue	FGFR1-38 (kcal/mol)	Residue	FGFR2-38 (kcal/mol)
Leu484	-2.11	Leu487	-3.21
Gln491	-1.34	Phe492	-3.35
Lys514	-3.94	Gly493	-1.51
Ile545	-1.59	Val495	-2.73
Glu562	-2.71	Ala515	-1.69
Tyr563	-2.19	Lys517	-3.62
Ala564	-2.25	Glu565	-2.55
Gly567	-1.16	Tyr566	-2.96
Leu630	-1.63	Ala567	-2.32
Ala640	-1.37	Gly570	-2.49
		Leu633	-3.17
